# Meteorological factors and its association with hand, foot and mouth disease in Southeast and East Asia areas: a meta-analysis

**DOI:** 10.1017/S0950268818003035

**Published:** 2018-11-19

**Authors:** Chunxiao Duan, Xuefeng Zhang, Hui Jin, Xiaoqing Cheng, Donglei Wang, Cangjun Bao, Minghao Zhou, Tauseef Ahmad, Jie Min

**Affiliations:** 1 Department of Epidemiology and Health Statistics, School of Public Health, Southeast University, Nanjing (210009), China; 2 Key Laboratory of Environmental Medicine Engineering, Ministry of Education, School of Public Health, Southeast University, Nanjing (210009), China; 3 Jiangsu Provincial Center for Disease Control and Prevention, China

**Keywords:** Hand, foot and mouth disease, meteorological factors, correlation coefficient, meta-analysis

## Abstract

Since the late 1990s, hand, foot and mouth disease (HFMD) has become a common health problem that mostly affects children and infants in Southeast and East Asia. Global climate change is considered to be one of the major risk factors for HFMD. This study aimed to assess the correlation between meteorological factors and HFMD in the Asia-Pacific region. PubMed, Web of Science, Embase, China National Knowledge Infrastructure, Wanfang Data and Weipu Database were searched to identify relevant articles published before May 2018. Data were collected and analysed using R software. We searched 2397 articles and identified 51 eligible papers in this study. The present study included eight meteorological factors; mean temperature, mean highest temperature, mean lowest temperature, rainfall, relative humidity and hours of sunshine were positively correlated with HFMD, with correlation coefficients (CORs) of 0.52 (95% confidence interval (CI) 0.42–0.60), 0.43 (95% CI 0.23–0.59), 0.43 (95% CI 0.23–0.60), 0.27 (95% CI 0.19–0.35), 0.19 (95% CI 0.02–0.35) and 0.19 (95% CI 0.11–0.27), respectively. There were sufficient data to support a negative correlation between mean pressure and HFMD (COR = −0.51, 95% CI −0.63 to −0.36). There was no notable correlation with wind speed (COR = 0.10, 95% CI −0.03 to 0.23). Our findings suggest that meteorological factors affect the incidence of HFMD to a certain extent.

## Introduction

Hand, foot and mouth disease (HFMD) is a common infectious disease caused by a group of enteroviruses, including coxsackievirus A16 (CA16) and enterovirus 71 (EV71) [1]. Since the late 1990s, HFMD has been a concern in Asia-Pacific countries [2], including China, Japan, South Korea, Vietnam and Singapore. In March 2008, an outbreak led to the death of 23 children infected with EV71 in Fuyang city, Anhui Province, China [3]. According to statistics from the National Health and Family Planning Commission regarding category C infectious diseases, there were 994 882 cases of paediatric HFMD reported in China in 2014, including 16 872 (1.69%) severe cases and 624 (0.06%) fatal cases [4]. From January 2000 to December 2015, there were 2 521 199 cases of HFMD reported in Japan. The majority of cases involved were children under the age of 5 years; this age group accounted for, and more than 80.4% of the reported cases from 2000 to 2014 [5]. In Vietnam, the number of reported cases in 2008 and 2009 was approximately 10 000, twice that of 2007. The outbreak of HFMD in Vietnam in 2012 resulted in 157 654 cases, and the incidence remained high in the following years [6]. In 2008, the largest ever outbreak in Singapore was reported, a total of 29 686 cases, with one case resulting in death [7]. HFMD caused a heavy economic and social burden, resulting in an important public health problem that seriously threatened the life and health of children and infants.

Although China pioneered the development of an innovative EV71 HFMD vaccine, this vaccine was only effective to prevent HFMD caused by EV71 infection, and could not prevent HFMD caused by infection with other enteroviruses (including CA16) [8]. Therefore, identification of risk factors for HFMD, and targeted prevention and control would be of assistance in reducing the incidence of HFMD. Currently, a large number of studies at home and abroad have focused on the impact of meteorological factors on the incidence of HFMD [9–11]. Analysis and comparison of the HFMD data with the corresponding meteorological data revealed a certain correlation between incidence of HFMD and temperature, humidity and air pressure. Although these studies were able to show that the incidence of HFMD was closely related to meteorological factors, the results of the studies were not consistent. For example, a study conducted by Zhao *et al*. [9] reported a positive correlation between mean temperature and HFMD incidence, while Wei *et al*. [12] reported a negative result. In another example, Chen *et al*. [13] found that the mean pressure and HFMD incidence was negatively correlated, while Du *et al*. [11] reported a positive correlation. Wei *et al*. also reported no significant correlation between mean pressure and the incidence of HFMD. These inconsistencies also existed with respect to other meteorological factors.

It was clear from previous studies that the correlation between meteorological factors and HFMD were not consistent. Therefore, the current study was conducted to perform a meta-analysis of published articles, with the aim of identifying meteorological risk factors for HFMD and the role of these risk factors in the pathogenesis of HFMD. The aim is to provide baseline information and a scientific basis for prevention and control measures of HFMD.

## Material and methods

### Search strategy

A literature search was performed using PubMed, Web of Science, Embase, China National Knowledge Infrastructure, Wanfang Data and Weipu Database. We used the following key words: hand foot and mouth disease (HFMD), meteorological, climate change, temperature, precipitation, air pressure, humidity, wind speed and sunshine. Articles published before May 2018 were included in the current study. References from the retrieved documents were also checked to include any additional relevant articles.

### Selection criteria

The inclusion criteria were as follows: (1) article language was Chinese or English; (2) study with a reported sample size; and (3) study provided clear correlation coefficient (COR), risk ratio (RR), odds ratio (OR) and incidence rate ratio (IRR) between meteorological factors and HFMD incidence.

Exclusion criteria were as follows: (1) review article; (2) repeatedly published papers; (3) articles with irrelevant data or lack of required information.

### Data collection and quality assessment

The first author, year of publication, title, location, study period, time sample size, meteorological factors and COR were recorded on a form. To evaluate the impact of study quality on the results of the study, we designed an assessment programme according to standard guidelines [14–16]. The evaluation scheme included nine items assessing reporting quality, external validity and bias, with possible scores ranging from 0 (poor quality) to 10 (high quality). Each document was independently scored by two researchers and discrepancies were resolved jointly [8].

### Statistical analysis

R software (R Foundation for Statistical Computing, Vienna, Austria) was used for meta-analysis. First, *Q* and *I*^2^ statistics were used to estimate heterogeneity among studies. According to the *Q*-statistic, if *P* value of *<*0.10 indicates heterogeneity in the risk factors among studies, then in such cases the random-effect model was used for the meta-analysis. Otherwise, the fixed-effect model was used. When we extracted data from the included literature, we found that most of the articles used Spearman correlation to analyse the relationship between meteorological factors and HFMD, for instance, Chen *et al*. [13], and the others used Pearson correlation to analyse this association, for example, Song *et al*. [17]. If the COR was not given in the article, we could extract the RR (risk ratio) [18–21], OR [22,23] and IRR (incidence rate ratio) [2] values. Among the Southeast Asian countries researched, the incidences were all <5% [2,20,21,23–25]. When the disease incidence is <5%, OR is an excellent approximation of RR, and IRR can be regarded as OR [26]. Then methods were available to convert the OR to COR [27]. The steps are as follows.

We converted from the log OR to the standardised mean difference (SMD) using
1
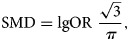

where *π* is the mathematical constant (approximately 3.14).

We then could convert from the standardised mean difference (SMD) to the correlation (*r* value) using
2
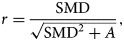

where *A* is the parameter related to the sample size (*n*_1_ and *n*_2_) of the two sets of data in the correlation analysis, where *n*_1_ ≠ *n*_2_
3
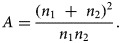


The parameter (*A*) depends on the ratio of *n*_1_ to *n*_2_, rather than the absolute values of these numbers. Therefore, if *n*_1_ and *n*_2_ are not sure, using *n*_1_ = *n*_2_, this will yield *A* = 4.

Summary statistics were then calculated, as most meta-analyses do not directly use CORs when combining CORs, because the variance of each COR is too dependent on the correlation [27–29]. The commonly used method is to calculate the sample COR (summary *r* value) of each study by Fisher's *Z* transform. We calculated Fisher's *Z* value and its standard error SE_*z*_ for analysis, which yield the summary effect (summary *Z*) and 95% confidence interval (CI). Then the summary *Z* value was transformed into the summary *r* value [29]. The formulas are as follows.

The transformation from sample COR (*r* value) to Fisher's *Z* value is given by
4
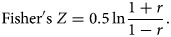


The standard error of *Z* is
5
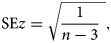

where *n* is the time sample size.

Then we convert each of summary *Z* values back to CORs (summary *r*) using
6
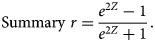


To calculated the summary *r*, and 95% CI. Based on the heterogeneity test results, we then judged the combination of effect values (summary *r*) using a random-effect model or a fixed-effect model. The hypothesis test was used to judge whether the correlation was statistically significant. The data were calculated and transformed using R software. Finally, forest plots were used to indicate the effect size. Publication bias was assessed with funnel plots and Egger's test.

## Results

### Characteristics of eligible studies

**Fig. 1.**
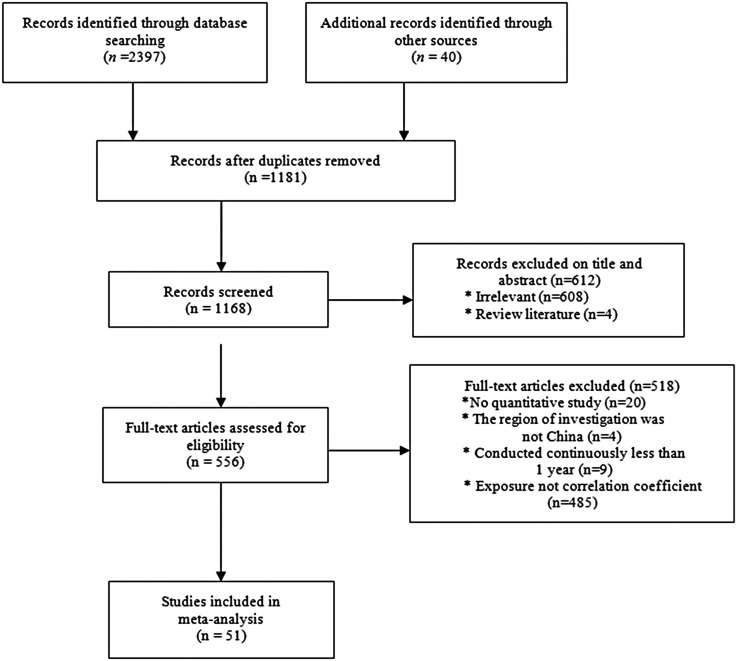
Flowchart of study selection.

A total of 2437 articles were retrieved and 51 articles were included in the present study (Fig. 1). Detailed information regarding the meta-analysis is provided in Tables 1 and 2.

**Table 1.** Characteristics of the 51 publications included in the meta-analysis

	Reference	Location	Study period	Time sample size	Statistical method	Resolution	Climate group
Tian *et al*. [18]		Beijing, China	2010–2012	36	Bayesian spatiotemporal Poisson regression model	Monthly	Temperate
Li *et al*. [30]		Shandong, China	2008	47	Spatiotemporal mixed model	Weekly	Temperate
Chen [13]		Mainland, China	2010–2014	60	Spearman correlation analysis	Monthly	
Zhang and Wang [31]		Hainan, China	2010–2014	60	Univariate and multivariate linear regression analyses	Monthly	Tropical
Du *et al*. [32]		Guangdong, China	2011–2014	208	Seasonal autoregressive integrated moving average (SARIMA) model	Weekly	Subtropical
Wu *et al*. [22]		Hunan, China	2009–2015	84	Spatial autocorrelation and spatiotemporal cluster analysis	Monthly	Subtropical
Gou *et al*. [19]		Gansu, China	2010	12	Bayesian spatial conditional autoregressive model	Monthly	Temperate
Phung *et al*. [20]		Vietnam	2011–2014	48	Generalised linear model (GLD)	Monthly	Tropical
Zhao *et al*. [9]		Huainan, China	2009–2014	312	Distributed lag non-linear model (DLNM)	Weekly	Subtropical
Du *et al*. [11]		Mainland, China	2011	12	Classification and regression tree model (CART)	Monthly	
Jiang *et al*. [33]		Qingdao, China	2007–2014	416	Spearman rank correlation analysis	Weekly	Temperate
Han [34]		Jinan, China	2007–2014	96	Spearman correlation analysis	Monthly	Temperate
Song *et al*. [17]		Zhengzhou, China	2010–2014	60	Pearson correlation analysis	Monthly	Temperate
Dong and Wang [35]		Xinzheng, China	2012–2015	48	Logistic regression	Monthly	Temperate
Li *et al*. [36]		Suizhou, China	2010–2014	60	Multiple linear regression	Monthly	Temperate
Zhou and Gao [37]		Shanghai, China	2010–2012	156	Multiple linear stepwise regression	Weekly	Subtropical
Wang *et al*. [10]		Qinzhou, China	2010–2015	72	Multiple linear stepwise regression	Monthly	Temperate
You *et al*. [36]		Kunming, China	2014	365	Multiple linear regression	Daily	Subtropical
Gu *et al*. [38]		Jiangyin, China	2009–2014	72	Pearson correlation analysis	Monthly	Subtropical
Xu *et al*. [10]		Jiayuguan, China	2008–2012	1825	DLNM	Daily	Temperate
Zhou *et al*. [39]		Chengdu, China	2011–2013	156	logistic regression model	Weekly	Subtropical
Lin *et al*. [40]		Hangzhou, China	2014	365	Pearson correlation analysis	Daily	Subtropical
Zhou and Yu [41]		Nanjing, China	2011–2014	48	Multiple linear stepwise regression	Monthly	Subtropical
Kim *et al*. [25]		South Korea	2010–2013	208	Generalised additive model (GAM)	Weekly	Subtropical
Wei *et al*. [42]		Shanxi, China	2009–2013	261	SARIMA model	Weekly	Temperate
Xu *et al*. [43]		Beijing, China	2010–2012	1095	DLNM	Daily	Temperate
Luo *et al*. [42]		Guangzhou, China	2009–2012	48	Concentration and circular distribution method	Monthly	Subtropical
Feng *et al*. [44]		Zhengzhou, China	2008–2016	312	SARIMA model	Weekly	Temperate
Xiang *et al*. [45]		Shanghai, China	2010–2013	208	Back-propagation neural network model	Weekly	Subtropical
Li *et al*. [46]		Beijing, China	2009–2013	60	Multivariate linear regression	Monthly	Temperate
Song *et al*. [47]		Guangzhou, China	2009–2013	252	SARIMA model	Weekly	Subtropical
Feng *et al*. [48]		Zhengzhou, China	2008–2012	234	SARIMA model	Weekly	Temperate
Chen *et al*. [49]		Suzhou, China	2012–2013	24	Spearman's rank correlation analysis	Monthly	Subtropical
Wu [50]		Laiwu, China	2010–2012	36	Series analysis	Monthly	Temperate
Wei and Zhang [12]		Linyi, China	2007–2012	72	Pearson correlation analysis	Monthly	Temperate
Shi *et al*. [51]		Laiwu, China	2011–2013	36	Linear regression	Monthly	Temperate
Wang *et al*. [52]		Kunming, China	2009–2012	36	Univariate and multivariate linear regression	Monthly	Subtropical
Bo *et al*. [23]		Mainland, China	2008–2009	12	Spatial autologistic regression model	Monthly	
Huang *et al*. [53]		Guangzhou, China	2008–2011	212	GAM	Weekly	Subtropical
Liu *et al*. [54]		Weifang, China	2007–2010	208	Univariate correlation and stepwise multiple regression analyses	Weekly	Temperate
Liu *et al*. [54]		Hebei, China	2009–2011	36	Multiple linear stepwise regression	Monthly	Subtropical
Zhuang *et al*. [55]		Shanghai, China	2005–2010	72	Pearson correlation analysis	Monthly	Subtropical
Tian *et al*. [56]		Baoji, China	2009–2011	156	Principal component analysis	Weekly	Temperate
Liu *et al*. [57]		Renqiu, China	2010–2012	36	Pearson correlation analysis	Monthly	Temperate
Luo *et al*. [58]		Guangzhou, China	2010–2011	24	Non-parametric correlation analysis	Monthly	Subtropical
Zheng *et al*. [59]		Shenzhen, China	2008–210	36	Geographically weighted regression model (GWR)	Monthly	Subtropical
Wang *et al*. [60]		Liaocheng, China	2009–2011	36	Pearson correlation analysis	Monthly	Temperate
Hu and Dong[61]		Wuwei, China	2008–2010	1095	Pearson correlation analysis	Daily	Temperate
Onozuka and Hashizume [21]		Japan	2000–2010	520	DLNMs	Weekly	Subtropical
Hii *et al*. [2]		Singapore	2001–2008	416	Time-series Poisson regression model	Weekly	Tropical
MA *et al*. [62]		Hong Kong, China	2000–2004	260	Spearman's rank correlation analysis	Weekly	Subtropical

**Table 2.** Summary of the studies included on the relationships between meteorological factors with HFMD

Reference	Location	Mean temperature (°C)	Mean maximum temperature (°C)	Mean minimum temperature (°C)	Mean air pressure（kPa）	Rainfall (mm)	Average relative humidity (%)	Hours of sunshine (hour)	Mean wind speed (m/s)
Tian *et al*. [18]	Beijing, China	0.01	n.a.	n.a.	n.a.	0.01	0.01	n.a.	n.a.
Li *et al*. [30]	Shandong, China	n.a.	0.14	0.14	−0.11	n.a.	0.07	n.a.	0.08
Chen *et al*. [13]	Mainland, China	0.71	n.a.	n.a.	−0.56	0.54	0.40	−0.09	n.a.
Zhang and Wang [31]	Hainan, China	0.90	n.a.	n.a.	−0.73	n.a.	n.a.	n.a.	−0.10
Du *et al*. [32]	Guangdong, China	0.66	n.a.	n.a.	n.a.	n.a.	n.a.	n.a.	n.a.
Wu *et al*. [22]	Hunan, China	0.17	n.a.	n.a.	n.a.	0.09	0.09	n.a.	n.a.
Gou *et al*. [19]	Gansu, China	0.08	n.a.	n.a.	n.a.	n.a.	−0.02	n.a.	n.a.
Phung *et al*. [20]	Vietnam	0.01	n.a.	n.a.	n.a.	0.01	0.00	n.a.	n.a.
Zhao *et al*. [9]	Huainan, China	0.34	n.a.	n.a.	−0.36	0.02*	−0.04*	n.a.	n.a.
Du *et al*. [11]	Mainland, China	0.49	n.a.	n.a.	0.26	n.a.	0.31	n.a.	n.a.
Jiang *et al*. [33]	Qingdao, China	0.77	n.a.	n.a.	n.a.	0.33	0.51	0.01*	n.a.
Han [34]	Jinan, China	0.72	n.a.	n.a.	−0.66	0.16	0.11	0.18	0.01*
Song *et al*. [17]	Zhengzhou, China	0.55	n.a.	n.a.	−0.56	0.26*	−0.04*	0.58	0.34
Dong and Wang [35]	Xinzheng, China	0.56	n.a.	n.a.	−0.15	0.48	0.15	−0.13	0.37
Li *et al*. [36]	Suizhou, China	0.50	n.a.	n.a.	−0.62	0.41	0.25*	0.31	n.a.
Zhou and Gao [37]	Shanghai, China	0.33	0.37	0.33	−0.44	0.16	0.31	n.a.	n.a.
Wang *et al*. [10]	Qinzhou, China	0.84	n.a.	n.a.	−0.73	0.42*	0.84	0.67	−0.62
You *et al*. [36]	Kunming, China	0.53	n.a.	n.a.	−0.42	0.20	0.06*	n.a.	−0.06*
Gu *et al*. [38]	Jiangyin, China	0.40	n.a.	n.a.	−0.49	0.20*	0.15*	0.04*	0.15*
Xu *et al*. [10]	Jiayuguan, China	0.25	n.a.	n.a.	−0.15	0.02*	−0.21	n.a.	0.13
Zhou *et al*. [39]	Chengdu, China	0.33	0.32	0.32	−0.26	0.21	−0.06*	n.a.	−0.02*
Lin *et al*. [40]	Hangzhou, China	0.85	n.a.	n.a.	n.a.	0.80	0.87	n.a.	n.a.
Zhou and Yu [41]	Nanjing, China	0.53	n.a.	n.a.	n.a.	0.58	n.a.	0.19*	n.a.
Kim *et al*. [25]	South Korea	0.61	n.a.	n.a.	n.a.	0.39	0.49	−0.15	n.a.
Wei *et al*. [42]	Shanxi, China	0.63	0.62	0.66	n.a.	0.26	0.19	0.26	n.a.
Xu *et al*. [43]	Beijing, China	0.83	n.a.	n.a.	n.a.	0.22	0.37	0.09	−0.01*
Luo *et al*. [42]	Guangzhou, China	0.47	n.a.	n.a.	n.a.	0.60	0.35	n.a.	n.a.
Feng *et al*. [44]	Zhengzhou, China	0.39	0.39	0.37	−0.43	0.16	−0.06*	0.34	0.26
Xiang *et al*. [45]	Shanghai, China	0.38	0.40	0.38	−0.49	0.11*	0.13	−0.04*	0.07*
Li *et al*. [46]	Beijing, China	0.71	0.71	0.71	−0.76	0.65	0.41	0.12*	−0.04*
Song *et al*. [47]	Guangzhou, China	0.17	−0.04	−0.22	n.a.	0.21	0.20	n.a.	0.05
Feng *et al*. [48]	Zhengzhou, China	0.65	0.63	0.62	−0.65	n.a.	−0.14	0.24	n.a.
Chen *et al*. [49]	Suzhou, China	0.57	n.a.	n.a.	n.a.	0.44	0.31	0.27	0.40
Wu [50]	Laiwu, China	0.57	n.a.	n.a.	−0.73	n.a.	0.66	0.50	0.04*
Wei and Zhang [12]	Linyi, China	−0.36	n.a.	n.a.	0.19*	−0.19*	−0.57	0.37	0.15*
Shi *et al*. [51]	Laiwu, China	0.58	0.60	0.80	n.a.	n.a.	n.a.	n.a.	n.a.
Wang *et al*. [52]	Kunming, China	0.58	n.a.	n.a.	−0.67	0.21*	0.36*	0.15*	0.25*
Bo *et al*. [23]	Mainland, China	0.04	n.a.	n.a.	n.a.	0.04	n.a.	n.a.	0.02
Huang *et al*. [53]	Guangzhou, China	0.28	n.a.	n.a.	n.a.	0.28	0.27	n.a.	0.16
Liu *et al*. [54]	Weifang, China	0.49	n.a.	n.a.	−0.15	0.15	0.09*	n.a.	−0.17
Liu *et al*. [54]	Hebei, China	0.61	n.a.	n.a.	−0.69	0.20*	n.a.	n.a.	0.39*
Zhuang *et al*. [55]	Shanghai, China	0.33	0.27	0.34	n.a.	0.14*	0.23*	0.05*	n.a.
Tian *et al*. [56]	Baoji, China	0.81	n.a.	n.a.	−0.74	0.57	n.a.	n.a.	n.a.
Liu *et al*. [57]	Renqiu, China	0.58	n.a.	n.a.	n.a.	n.a.	0.51	n.a.	n.a.
Luo *et al*. [58]	Guangzhou, China	0.56	n.a.	n.a.	n.a.	0.68	0.44	0.19*	n.a.
Zheng *et al*. [59]	Shenzhen, China	0.26	n.a.	n.a.	n.a.	−0.30	n.a.	n.a.	n.a.
Wang *et al*. [60]	Liaocheng, China	0.51	0.52	0.47	−0.57	0.12*	0.10*	0.50	−0.07*
Hu and Dong [61]	Wuwei, China	0.79	0.79	0.77	−0.87	0.34*	−0.81	n.a.	0.67
Onozuka and Hashizume [21]	Japan	0.01	n.a.	n.a.	n.a.	n.a.	0.01	n.a.	n.a.
Hii *et al*. [2]	Singapore	n.a.	0.02	−0.01	n.a.	0.01	n.a.	n.a.	n.a.
Ma *et al*. [62]	Hong Kong, China	0.26	0.26	0.24	−0.33	0.23	0.14	−0.01*	0.01*

n.a., Data were not searched; *no statistical significance, *P* > 0.05.

All studies shown in Tables 1 and 2 were conducted in the Asia-Pacific areas; the time unit was month, week and day. The number of included studies were as follows: mean temperature (49 studies), mean maximum temperature (15 studies), mean minimum temperature (15 studies), mean air pressure (28 studies), rainfall (41 studies), average relative humidity (42 studies), hours of sunshine (24 studies) and mean wind speed (25 studies).

### Correlation between meteorological factors and HFMD

**Fig. 2.**
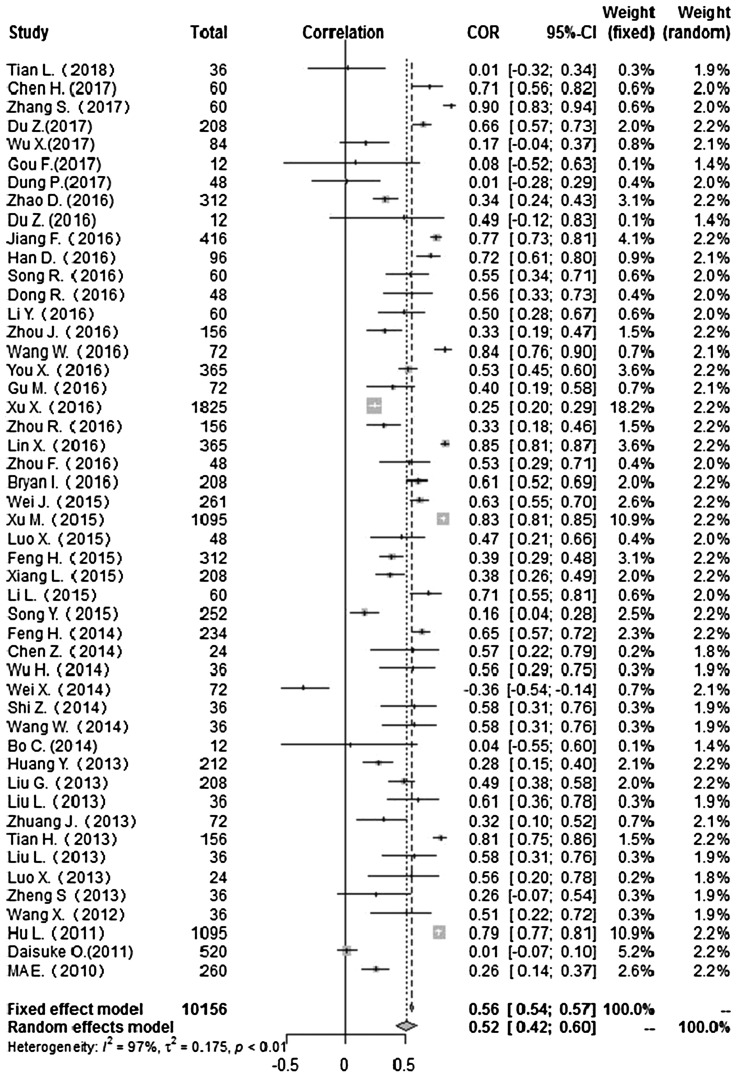
Forest plot of the correlation between mean temperature and incidence of HFMD. COR, correlation coefficient; CI, confidence interval.

**Fig. 3.**
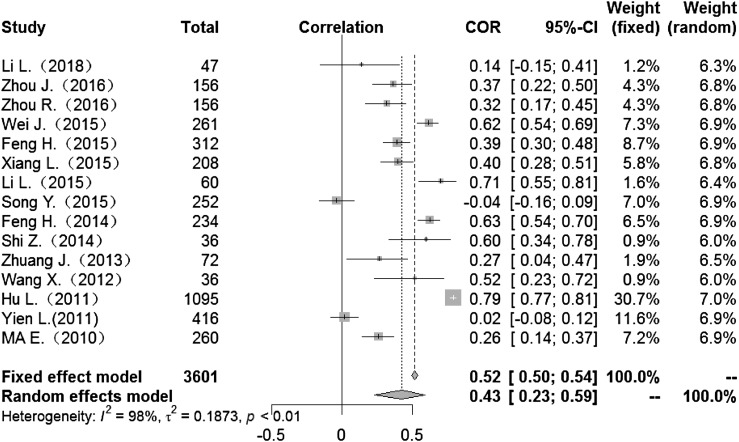
Forest plot of the correlation between mean maximum temperature and incidence of HFMD. COR, correlation coefficient; CI, confidence interval.

**Fig. 4.**
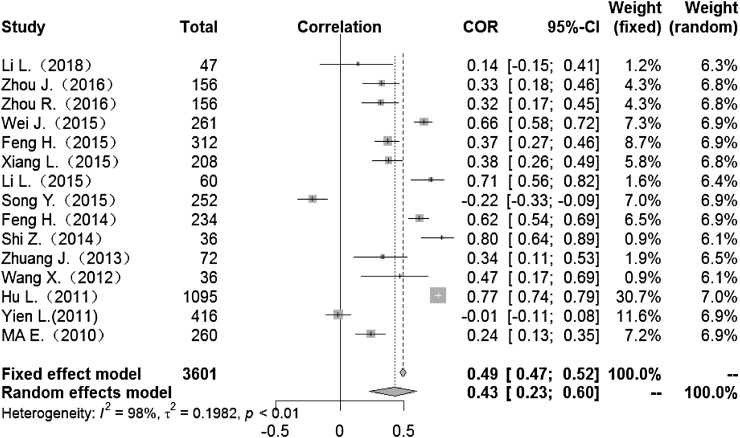
Forest plot of the correlation between mean minimum temperature and incidence of HFMD. COR, correlation coefficient; CI, confidence interval.

**Fig. 5.**
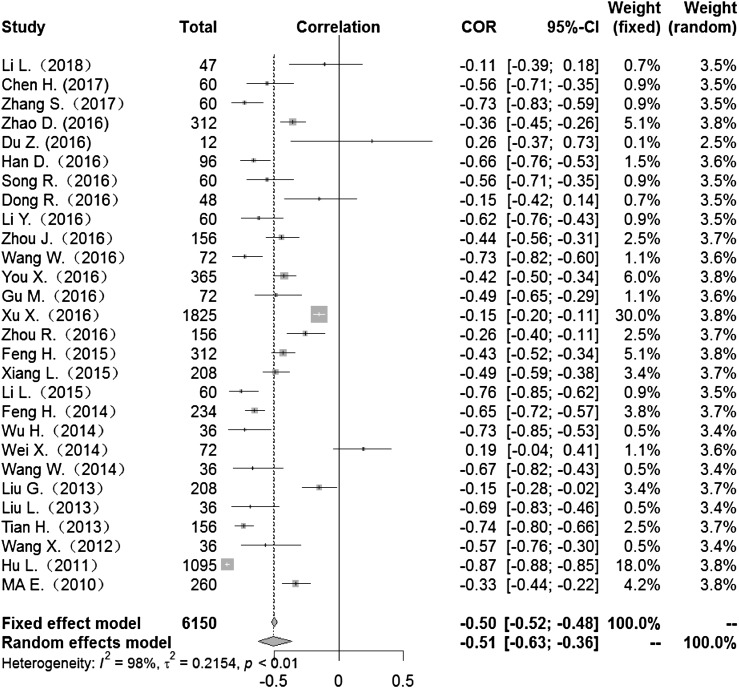
Forest plot of the correlation between mean air pressure and incidence of HFMD. COR, correlation coefficient; CI, confidence interval.

**Fig. 6.**
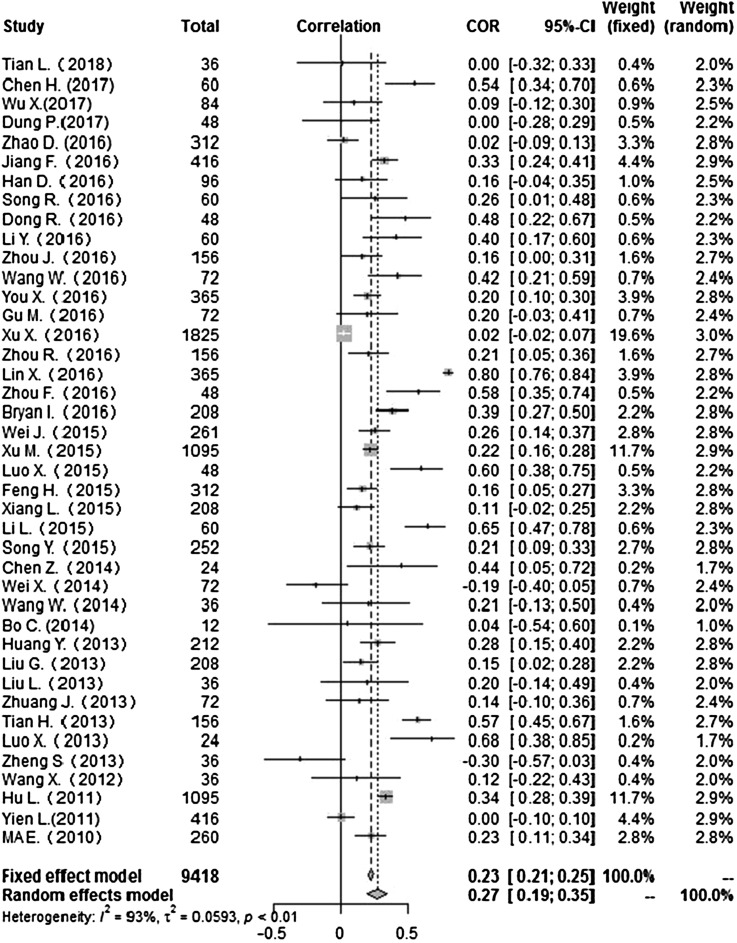
Forest plot of the correlation between rainfall and incidence of HFMD. COR, correlation coefficient; CI, confidence interval.

**Fig. 7.**
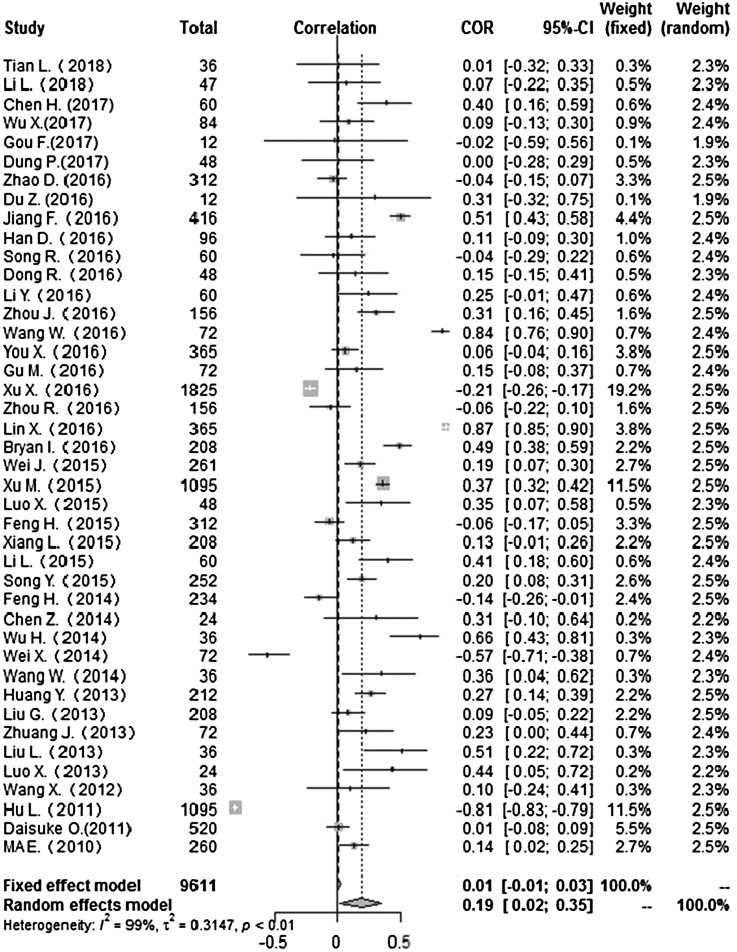
Forest plot of the correlation between mean relative humidity and incidence of HFMD. COR, correlation coefficient; CI, confidence interval.

**Fig. 8.**
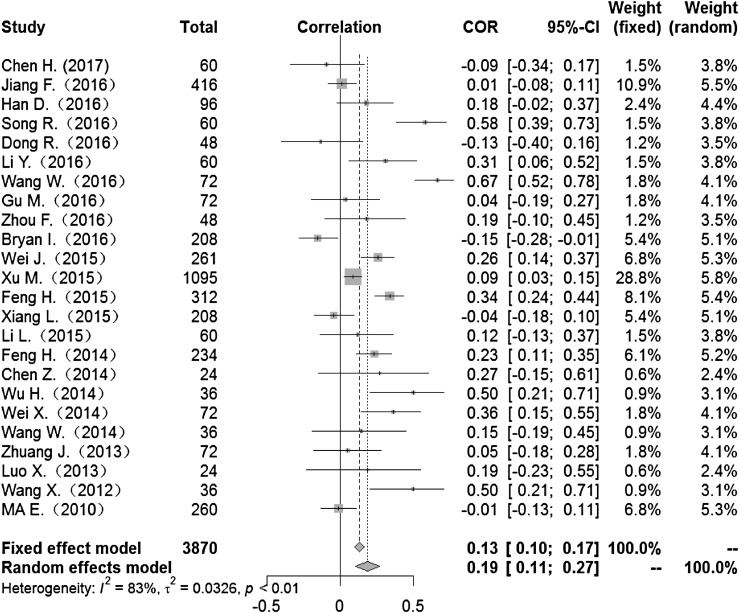
Forest plot of the correlation between hours of sunshine and incidence of HFMD. COR, correlation coefficient; CI, confidence interval.

**Fig. 9.**
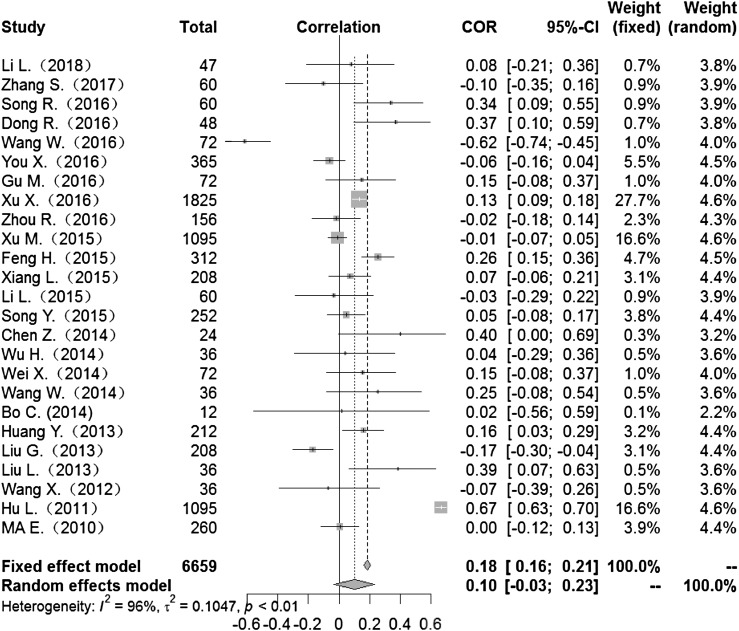
Forest plot of the correlation between mean wind speed and incidence of HFMD. COR, correlation coefficient; CI, confidence interval.

Heterogeneity test: mean temperature (*I*^2^ = 97%, *P* < 0. 001) suggested that there was heterogeneity, using the random-effects model to pool the effect values. The combined effect showed that the correlation between mean temperature and HFMD was statistically significant. CORs (mean temperature, mean air pressure and average wind speed with HFMD) were 0.52 (95% CI 0.42–0. 60), −0.51(95% CI −0.63 to −0.36) and 0.10 (95% CI −0.03 to 0.23) (Figs 2, 5, 9), the other CORs were displayed in the diagram (Figs 3, 4, 6, 7, 8), respectively, indicating that mean temperature and mean air pressure were correlated with HFMD.

The forest plots of other meteorological factors can be found in Table 3. The results of heterogeneity testing demonstrated statistically significant heterogeneity with respect to mean temperature, average maximum temperature, average minimum temperature, mean air pressure, rainfall, average relative humidity, sunshine hours and average wind speed, using a random-effects model to merge effect values (Table 3).

**Table 3.** Meta-analysis of the correlation between meteorological factors and HFMD

Meteorological factors	No. of studies	*I*^2^ (*P*-value)	*COR* (95%CI)
Mean temperature (°C)	49	97% (*P* = 0.000)	0.52 (0.42–0.60)
Mean maximum temperature (°C)	15	98% (*P* = 0.000)	0.43 (0.23–0.59)
Mean minimum temperature (°C)	15	98% (*P* = 0.000)	0.43 (0.23–0.60)
Mean air pressure（kPa）	28	98% (*P* = 0.000)	−0.51 (−0.63 to −0.36)
Rainfall (mm)	41	93% (*P* = 0.000)	0.27 (0.19–0.35)
Average relative humidity (%)	42	99% (*P* = 0.000)	0.19 (0.02–0.35)
Hours of sunshine (hour)	24	93% (*P* = 0.000)	0.19 (0.11–0.27)
Mean wind speed (m/s)	25	96% (*P* = 0.000)	0.10 (−0.03 to 0.23)*

*No statistical significance, *P* > 0.05; COR, correlation coefficient.

### Subgroup analysis

The study also included subgroup analysis. In Table 4, the studies of daily resolution show that CORs of mean temperature, mean maximum temperature, mean minimum temperature, mean air pressure, rainfall and mean wind speed were highest compared with those of the remaining two groups, and the CORs of these factors were 0.70 (0.40–0.86), 0.79 (0.77–0.81), 0.77 (0.74–0.79), −0.57 (−0.89 to −0.16), 0.36 (0.09–0.58) and 0.21 (−0.17 to 0.54), respectively. In the subgroup analysis of humidity and hours of sunshine, the CORs in the subgroup of the month were 0.25 (0.09–0.41) and 0.21 (−0.17 to 0.54) higher than the values of the other groups.

**Table 4.** Subgroup analysis of the correlation between meteorological factors and HFMD (time resolution)

Meteorological factors	No. of studies	Month	No. of studies	Week	No. of studies	Day
Mean temperature (°C)	28	0.50 (0.37–0.61)	16	0.48 (0.34–0.59)	5	0.70 (0.40–0.86)
Mean maximum temperature (°C)	4	0.54 (0.30–0.71)	10	0.33 (0.17–0.48)	1	0.79 (0.77–0.81)
Mean minimum temperature (°C)	4	0.61 (0.35–0.78)	10	0.31 (0.11–0.48)	1	0.77 (0.74–0.79)
Mean air pressure（kPa）	15	−0.56 (−0.67 to −0.42)	10	−0.43 (−0.54 to −0.30)	3	−0.57 (−0.89 to −0.16)
Rainfall (mm)	22	0.28 (0.16–0.39)	14	0.22 (0.14–0.30)	5	0.36 (0.09–0.58)
Average relative humidity (%)	22	0.25 (0.09–0.41)	15	0.15 (0.03, 0.26)	5	0.09(−0.55 to 0.66)*
Hours of sunshine (hour)	16	0.26 (0.13–0.38)	7	0.10 (−0.04 to 0.23)*	1	0.09 (0.03–0.15)
Mean wind speed (m/s)	13	0.09 (−0.10 to 0.28)*	8	0.06 (−0.04 to 0.15)*	4	0.21 (−0.17 to 0.54)*

*No statistical significance, *P* > 0.05.

In subgroup analysis by regional climate, the studies of subtropical climate showed a slightly lower COR than the non-stratified group in relation to all the meteorological factors except average relative humidity, and the association of hours of sunshine with HFMD was not statistically significant and showed less heterogeneity (*I*^2^ = 19.7%, *P* = 0.003). The studies of tropical climate found no statistically significant correlation between the incidence of HFMD and any of the meteorological factors. The results of subgroup analysis based on exposed time resolution (climate group) were consistent with those without stratification, as shown in Table 4 (Table 5).

**Table 5.** Subgroup analysis of the correlation between meteorological factors and HFMD (regional climate)

Meteorological factors	No. of studies	Subtropical climate	No. of studies	Temperate climate	No. of studies	Tropical climate
Mean temperature (°C)	22	0.44 (0.31–0.55)	22	0.58 (0.44–0.69)	2	0.62 (−0.59 to 0.97)*
Mean maximum temperature (°C)	6	0.26 (0.12–0.40)	8	0.58 (0.40–0.72)	1	0.02 (−0.08 to 0.12)*
Mean minimum temperature (°C)	6	0.23 (0.03–0.42)	8	0.60 (0.44–0.73)	1	−0.01 (−0.11 to 0.08)*
Mean air pressure (kPa)	9	−0.43 (−0.50 to −0.36)	16	−0.53 (−0.71 to −0.34)	1	−0.73 (−0.83 to −0.59)
Rainfall (mm)	20	0.29 (0.15–0.43)	16	0.26 (0.16–0.35)	2	0.00 (−0.09 to 0.09)*
Average relative humidity (%)	18	0.26 (0.07–0.44)	21	0.12 (−0.14 to 0.37)*	1	0.00 (−0.28 to 0.29)*
Hours of sunshine (hour)	9	0.00 (−0.08 to 0.08)*	14	0.29 (0.18–0.39)	n.a.	n.a.
Mean wind speed (m/s)	10	0.08 (0.00–0.15)	13	0.10 (−0.12 to 0.30)*	1	−0.10 (−0.35 to 0.16)*

*No statistical significance, *P* > 0.05; n.a., data were not searched.

### Sensitivity analysis and publication bias

**Fig. 10.**
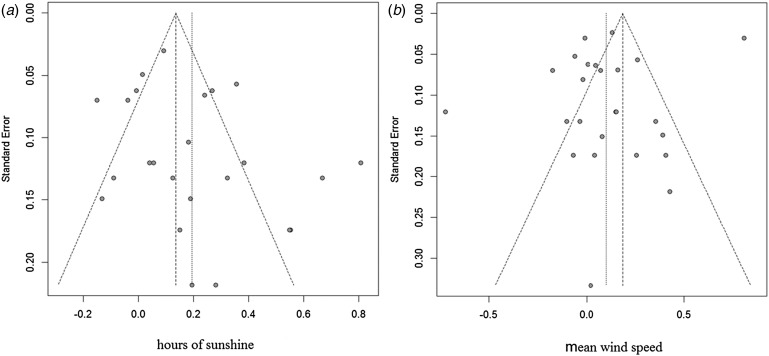
Funnel plots of hours of sunshine and mean wind speed.

**Fig. 11.**
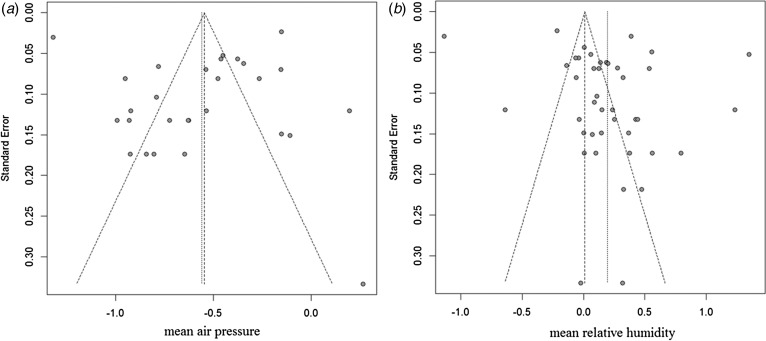
Funnel plots of mean air pressure and mean relative humidity.

**Fig. 12.**
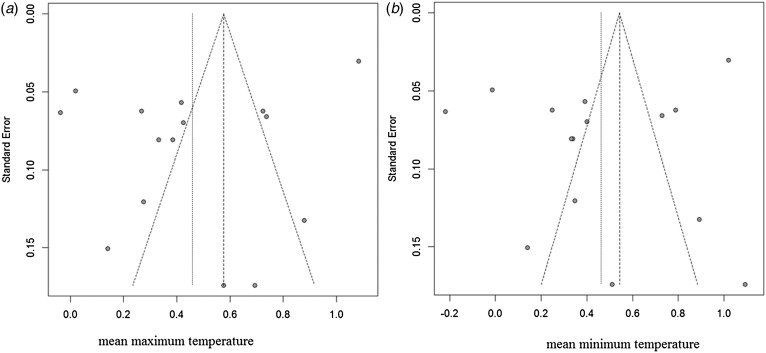
Funnel plots of mean maximum temperature and mean minimum temperature.

**Fig. 13.**
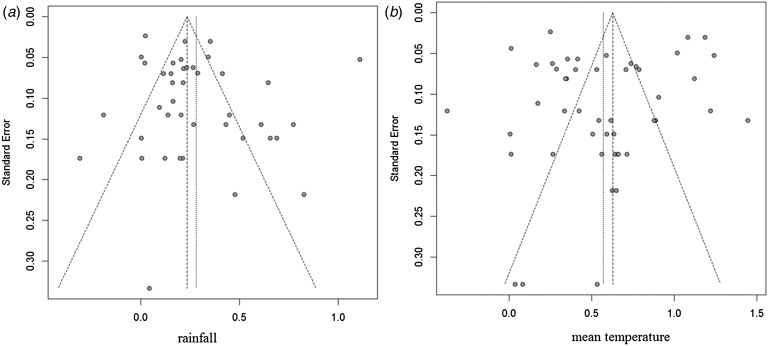
Funnel plots of rainfall and mean temperature.

Sensitivity analyses were performed to evaluate the effect of each study on the pooled results by excluding single studies sequentially. The findings showed that the stability of results not significantly differ after exclusion of individual studies. Funnel plot asymmetry was observed for studies of rainfall and mean temperature (Fig. 13). The funnel plots of other meteorological factors are shown in Table 6 (Figs 10–12). Egger's test was used to assess funnel plot asymmetry, as shown in Table 6. No publication bias existed in the meta-analysis.

**Table 6.** The publication bias of meteorological factors

Meteorological factors	Egger's test	
	*P*-value	*t-*Value
Mean temperature (°C)	0.51	−0.66
Mean maximum temperature (°C)	0.12	−1.64
Mean minimum temperature (°C)	0.23	−1.25
Mean air pressure (kPa)	0.84	−0.20
Rainfall (mm)	0.25	1.17
Average relative humidity (%)	0.05	2.01
Hours of sunshine (hour)	0.12	1.60
Mean wind speed (m/	0.29	−1.90

## Discussion

The current study found associations between meteorological factors and HFMD and indicated that these factors play an important role in the occurrence of HFMD. The COR for mean temperature was 0.52 (95% CI 0.42–0.60), that for mean maximum temperature was 0.43 (95% CI 0.23–0.59). CORs for mean minimum temperature, rainfall, mean relative humidity and sunshine were 0.43 (95% CI 0.23–0.60), 0.27 (95% CI 0.19–0.35), 0.19 (95% CI 0.02–0.35) and 0.19 (95% CI 0.11–0.27), respectively. These meteorological factors showed positive correlations with HFMD. However, mean air pressure was negatively correlated with HFMD, while mean wind speed showed no significant association (COR 0.10; 95% CI −0.03 to 0.23). The mechanism of meteorological factors on HFMD remains unclear. Possible reasons to explain these findings may be that, first, elevated temperatures contribute to the survival, reproduction and transmission of enterovirus in the outside environment [63]. There is also a threshold for the influence of temperature on the incidence of foot and mouth disease [5]. With increases in relative humidity and total rainfall, HFMD-causative pathogens are more likely to adhere to the surfaces of objects in the environment, and the probability of human contact with infectious agents increases [64, 65]. Decreases in air pressure result in lowered immunity in humans, thus increasing the risk of disease [66]. With decreases in the duration of sunshine, the time people spend in outdoor activities is reduced, thus reducing the chance of patient contacts [67]. Although the mechanism is not clear, our study examines the association between weather factors and HFMD, indicating that weather factors affect the incidence of HFMD infection.

The results of this study were consistent with the majority of research findings. However, a study conducted by Wei *et al*. [12] reported that the average temperature had a negative correlation with HFMD incidence (*r* = −0.36, *P* = 0.005), a finding that might be accounted for by a gap between the star time of the study and the establishment and improvement of the HFMD surveillance system [68]. In addition, most of the results of Wang *et al*. [52] were not statistically significant. Therefore, we should be cautious in interpretation of this and other studies to prevent over-generalisation and drawing the wrong conclusion. There was no significant correlation in the present study between the average wind speed and the incidence of HFMD; although this finding was consistent with the conclusion of most of the other studies, the COR was small and conclusions should not be arbitrarily drawn.

In subgroup analysis, no statistical significance was reported between mean relative humidity and subgroup of days. The same pattern was reported for hours of sunshine. It is possible that different time scales have an impact on the results of the study; while on the other hand, the effect was non-significant among the larger sample size studies. On the time scale, there was a lag effect in the subgroup of weeks, and there were seasonal problems in the monthly subgroup, all of which should be considered. It was concluded that time scales might be the factors affecting heterogeneity. As shown in Table 5, we found statistically significant association between meteorological factors and incidence of HFMD in subtropical and temperate regions. The subtropical and temperate climates are considered as those that are more suitable for the survival and reproduction of enteroviruses. Individuals living in these climates may participate in more outdoor activities, thereby increasing their exposure to pathogens [69, 70]. In the tropics, due to the existence of threshold, the excessive temperature may inhibit the survival and reproduction of the virus, thus reducing the opportunity for disease infection [5, 63].

The strength of our study is based on its design as a meta-analysis; this was the first meta-analysis to examine correlations between eight meteorological factors and the incidence of HFMD. We observed that some meteorological factors such as temperature, air pressure, duration of sunshine, humidity and rainfall might be risk factors for HFMD and confirmed that these meteorological factors might affect the incidence of HFMD to a certain extent, more reliable and power than the conclusions obtained from single studies. As shown in Table 6, we found that publication bias was not statistically significant.

The study has some limitations. First, we found the significant heterogeneity between the studies included in this meta-analysis. All of the included studies were conducted in Southeast and East Asia, especially in China. So differences in regional and analytical methods might result in high heterogeneity in estimates from the literature. Most of the included studies that were conducted in subgroup analysis only analysed time resolution and regional climate. Additional possibilities such as national income should be considered to better understand sources of heterogeneity. The second limitation is that we only considered the eight meteorological factors affecting HFMD, ignoring other factors. In the present study, there were few studies of some of the meteorological factors involved, such as mean maximum temperature, so the results might be biased. Confirmation and clarification of these findings will require larger sample size and wider research region, such as the studies reviewed were from other Asian countries or throughout the world. Lastly, the studies we examined in this meta-analysis were studies of association. The researches we included were cross-sectional designs that limited the causal inference. The true relationship between meteorological factors and HFMD could be more complex, and there are potential difficulties in accurately measuring complex associations. Therefore, the association between meteorological factors and HFMD might be one of many influencing factors.

In summary, among the eight meteorological factors examined, the average temperature, average maximum temperature, mean minimum temperature, mean air pressure, rainfall, mean relative humidity and sunshine were related to HFMD, indicating that these factors play important roles in the incidence of HFMD. However, only eight major meteorological factors were analysed in this study. HFMD is a multifactorial disease that may be affected by additional meteorological factors (such as evaporation, water vapour pressure and radiation). Further analyses should examine various comprehensive indicators.

In conclusion, the results of this meta-analysis provide epidemiological evidence that meteorological factors (such as temperature and air pressure) may increase the incidence of HFMD in the Asia-Pacific regions. Further research should be performed to explain clearly the correlation between meteorological factors and HFMD in other areas of the world, outside the Asia-Pacific region. At the same time, monitoring these meteorological factors would play a warning role in the occurrence and prevalence of HFMD and could provide information useful in the development of prevention and control measures for HFMD, particularly in subtropical and temperate climates.
